# Timing of parathyroidectomy in kidney transplant candidates: a systematic review and a proposed clinical algorithm

**DOI:** 10.3389/fendo.2026.1871106

**Published:** 2026-07-03

**Authors:** Yushuai Zhang, Lihong Song, Xuehai Bian, Yishen Zhao, Yutang Miao

**Affiliations:** 1 Department of Thyroid and Breast Surgery, Qilu Hospital of Shandong University Dezhou Hospital, Dezhou, China; 2 Nursing Department, Qilu Hospital of Shandong University Dezhou Hospital, Dezhou, China; 3 Department of Thyroid Surgery, China-Japan Union Hospital of Jilin University, Jilin Provincial Key Laboratory of Translational Medicine in Surgery, Jilin Provincial Engineering, Changchun, Jilin, China; 4 Laboratory of Thyroid Disease Prevention and Treatment Changchun, Changchun, Jilin, China

**Keywords:** chronic kidney disease, end-stage kidney disease, kidney transplantation, parathyroidectomy, secondary hyperparathyroidism

## Abstract

**Objective:**

Parathyroidectomy (PTx) is widely used for severe secondary hyperparathyroidism (SHPT) in kidney transplant candidates, but the optimal timing (pre-transplant versus post-transplant) remains controversial. This systematic review aimed to compare the outcomes of pre-transplant PTx and post-transplant PTx on graft loss, tertiary hyperparathyroidism (THPT), renal function, and complications, and to propose a clinical algorithm for PTx timing.

**Methods:**

We systematically searched PubMed, Embase, Web of Science, and Cochrane Library from inception to July 2024, with an update in April 2026. Studies directly comparing pre-transplant and post-transplant PTx in adult kidney transplant candidates were included. Notably, all eligible studies were observational, and no relevant interventional studies have been published to date. Due to heterogeneity in outcome definitions and measurement methods, no meta-analysis was performed; results were synthesized narratively and summarized in tables. Risk of bias was assessed using the Newcastle-Ottawa Scale.

**Results:**

Eight studies (4355 patients) were included. Graft loss events were low in both groups, with most studies indicating a potential trend toward lower graft loss in the pre-transplant PTx group; one study reported a significant adjusted odds ratio favoring pre-transplant PTx (0.547, 95% CI: 0.327–0.913). Pre-transplant PTx was associated with lower 1-month eGFR in one study, but this difference was absent at 36 months, and no long-term differences in eGFR or serum creatinine were observed across studies. THPT definitions varied, with no consistent between-group difference. Postoperative hypocalcemia rates were inconsistent. A large database study reported that pre-transplant PTx was associated with significantly higher 30-day composite morbidity, major adverse cardiovascular events, and readmission, with no difference in mortality. Based on the synthesized evidence, we propose a risk-stratified algorithm incorporating waitlist time, PTH level, dialysis vintage, and a validated nomogram to guide PTx timing.

**Conclusion:**

Pre-transplant PTx may be associated with lower graft loss and comparable long-term renal function compared with post-transplant PTx. Given the limitations of the current evidence, a personalized approach using the proposed algorithm may optimize clinical decision-making. Prospective studies are needed to validate this framework.

**Systematic review registration:**

https://www.crd.york.ac.uk/PROSPERO/view/CRD42024556524, identifier CRD42024556524.

## 1 Introduction

Chronic kidney disease (CKD) affects 10–15% of the global population, and a substantial proportion progresses ESKD requiring renal replacement therapy (1). Secondary hyperparathyroidism (SHPT) develops in approximately 50% of patients with advanced CKD and worsens with longer dialysis vintage (2–4). SHPT is strongly associated with fractures, cardiovascular events, and neuropsychiatric disorders, negatively impacting quality of life and survival (5, 6).

Kidney transplantation (KTx) is the most effective renal replacement therapy and can ameliorate SHPT. However, over 70% of KTx recipients develop persistent hyperparathyroidism within three months after transplantation (7), and this proportion rises to more than 80% within the first post-transplant year (8), partly because KTx corrects the uremic milieu but does not reverse established nodular parathyroid hyperplasia. However, more than 80% of KTx recipients develop persistent hyperparathyroidism within the first year post-transplant (8), partly because KTx corrects the uremic milieu but does not reverse established nodular parathyroid hyperplasia. Persistent hyperparathyroidism after KTx, termed tertiary hyperparathyroidism (THPT), is associated with graft dysfunction and loss (5, 6, 9). Parathyroidectomy (PTx) is the definitive treatment for refractory SHPT and THPT (10, 11).

Since the introduction of calcimimetics, the management of SHPT has shifted toward pharmacotherapy, with a declining trend in PTx rates among US dialysis patients (12) (13). However, cinacalcet use before KTx may mask nodular hyperplasia and increase the risk of post-transplant THPT (14, 15). In contrast, PTx as a more aggressive approach may offer better short- and long-term benefits for KTx candidates. Theoretically, pre-transplant PTx may effectively control severe SHPT and reduce the risk of post-transplant THPT and allograft injury, while it carries additional perioperative surgical risks for dialysis patients. By contrast, post-transplant PTx avoids surgical interference during the waiting period for kidney transplantation, but residual parathyroid lesions may persist and compromise long-term allograft function. Given the current limited evidence, neither surgical strategy can be regarded as inherently superior. Nevertheless, the optimal timing of PTx relative to KTx—whether before or after transplantation—remains controversial, with inconsistent and conflicting evidence.

Several narrative reviews (16–18) have summarized the general principles of PTx timing, but a systematic synthesis directly comparing pre- and post-transplant PTx is lacking. A recent literature review (19) concluded that early PTx is beneficial, drawing on clinical, surgical, and economic evidence. However, it did not quantitatively compare graft outcomes between the two timing strategies, nor did it specifically focus on the impact of waitlist time differences across countries. Moreover, no existing review has integrated guideline recommendations or provided a structured framework for clinical decision-making that incorporates individual patient risk stratification.

Therefore, this systematic review aimed to directly compare pre-transplant PTx with post-transplant PTx in kidney transplant candidates with SHPT, focusing on graft loss, THPT incidence, and renal function outcomes. We also summarize international guidelines and propose an exploratory clinical algorithm incorporating waitlist time, PTH level, dialysis vintage, and a validated nomogram to guide PTx timing. Our findings are intended to provide actionable evidence for clinicians managing this high-risk population.

## 2 Methods

This systematic review was conducted in accordance with the Preferred Reporting Items for Systematic Reviews and Meta-Analyses (PRISMA) 2020 statement (20). The study protocol was registered in PROSPERO (CRD42024556524) in July 2024 and amended in April 2026. The registered protocol was consistent with the final analysis; no major deviations occurred. The amendment in April 2026 only updated search dates without changing objectives or methods. This study did not involve individual patients and therefore did not require ethics committee approval.

### 2.1 Search strategy

We systematically searched PubMed, Embase, Web of Science, and Cochrane Library from database inception to July 2024, using MeSH terms and entry terms for “SHPT” and “KTx”. The search was updated in April 2026 using the same strategies with a date restriction of August 2024 to April 2026. We also manually screened reference lists of included studies and relevant reviews. Only English-language articles were considered.

### 2.2 Selection criteria

Studies were included if they: (i) were randomized controlled trials (RCTs), cohort studies, or case-control studies; (ii) directly compared PTx performed before KTx versus after KTx in adult (≥18 years) patients with ESKD and SHPT or THPT; (iii) reported at least one of the following outcomes: graft loss, tertiary hyperparathyroidism (THPT), estimated glomerular filtration rate (eGFR), delayed graft function (DGF), or postoperative complications (hypocalcemia, hoarseness, hematoma); and (iv) were published in English.

Exclusion criteria were: (i) letters, case reports, reviews, commentaries, or editorials; (ii) studies with incomplete or non-extractable data; (iii) duplicate publications; (iv) inaccessible full text; (v) pediatric studies (<18 years).

### 2.3 Data extraction

Two reviewers independently extracted data using a standardized form. Disagreements were resolved by consensus or a third reviewer. Extracted information included: first author, year, country, study period, study design, number of centers, sample size, patient demographics (age, sex, dialysis vintage), donor type (living/deceased), PTx surgical technique (subtotal PTx, total PTx with or without autotransplantation (AT)), timing of PTx relative to KTx, follow-up duration, and outcome data. For each outcome, we extracted the number of events and total patients (dichotomous outcomes) or means, standard deviations (SDs), and sample sizes (continuous outcomes). Where SDs were not reported, we calculated them from confidence intervals, standard errors, or interquartile ranges using the method of Wan et al. (21), which allows estimation of means and SDs from medians and interquartile ranges. When data were presented only as medians and ranges, we also applied the same method.

### 2.4 Risk of bias assessment

Two reviewers independently assessed the methodological quality of included studies using the Newcastle-Ottawa Scale (NOS) for cohort studies (22). The NOS evaluates three domains: selection of participants (0–4 stars), comparability of groups (0–2 stars), and ascertainment of outcome (0–3 stars). A study was considered good quality if it scored ≥7 stars, fair if 5–6 stars, and poor if ≤4 stars. Disagreements were resolved by discussion.

### 2.5 Outcome measures

**The primary outcomes were:**

Graft loss: return to long-term dialysis or retransplantation.

THPT: persistent hypercalcemia (corrected serum calcium >10.2 mg/dL or >2.55 mmol/L) with elevated PTH (>150 pg/mL or >16 pmol/L) occurring at least 6 months after KTx, as defined by each study. Where studies used different thresholds, we accepted the authors’ definition.

**Secondary outcomes included:**

eGFR at 1 year post-KTx (mL/min/1.73 m²)

Delayed graft function (DGF, need for dialysis within first week post-KTx)

Postoperative hypocalcemia (as defined by each study)

Other complications (hoarseness, hematoma, hungry bone syndrome)

### 2.6 Data synthesis

Due to substantial heterogeneity in outcome definitions (especially THPT), different follow-up time points, inconsistent PTH assay kits, and varying surgical techniques across studies, meta-analysis was deemed inappropriate and could lead to misleading pooled estimates. Therefore, a narrative synthesis was performed in accordance with PRISMA 2020 guidance for highly heterogeneous evidence.

## 3 Results

### 3.1 Study selection

**Figure 1:**
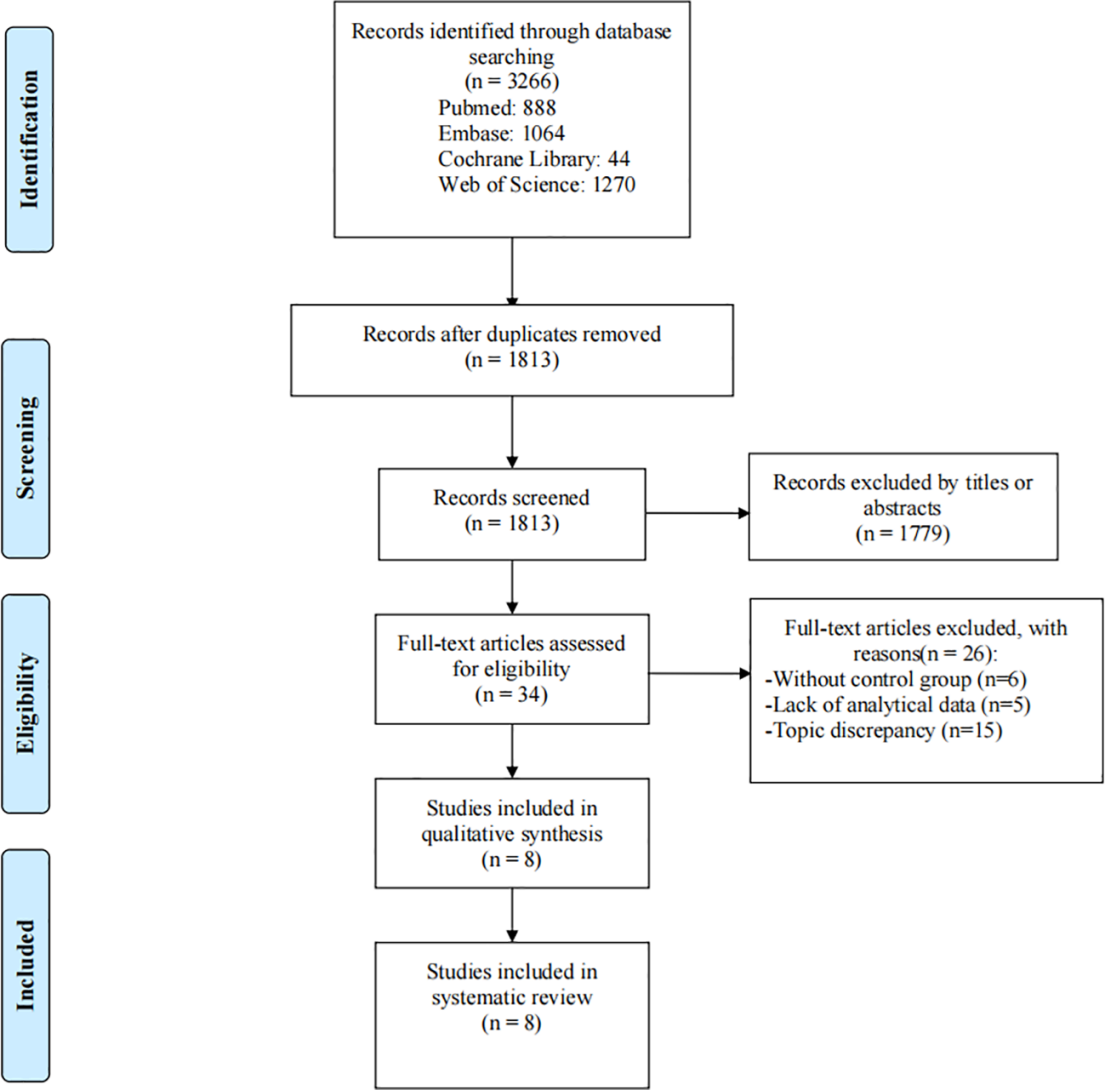
PRISMA flow diagram of literature research.

A systematic search identified 3266 records. After duplicate removal, 1813 records remained. After screening titles and abstracts, 1779 records were excluded, leaving 34 full-text articles to be assessed for eligibility. Twenty-six were excluded (reviews, no full text, incomplete data, or lack of direct pre- vs. post-transplant PTx comparison). Finally, eight eligible studies (including two separate cohorts from Littbarski et al. (23)) encompassing 4355 patients were included (5, 23–29). The detailed literature screening process is presented in the PRISMA flow diagram (**Figure 1**).

### 3.2 Study characteristics

All included studies were retrospective cohort studies published between 2012 and 2023, conducted in six countries (Korea, Germany, USA, Turkey, Netherlands, Japan). Sample sizes ranged from 66 to 2513 patients; follow-up ranged from 1 month to 7 years. Detailed characteristics are presented in **Table 1**.

**Table 1:** Characteristics of included studies.

First author, year	Country	Study design	Centers	Sample size (pre/post)	Follow-up	Main outcomes	NOS score
Callender 2017 (5)	USA	Retrospective cohort	Single	57/18	1 year	Graft loss, complications	8
Jeon 2012 (23)	Korea	Retrospective cohort	Multicenter	37/63	5 years	eGFR, THPT	7
Littbarski 2018 (short-term) (22)	Germany	Retrospective cohort	Single	52/14	1 year	KDIGO stage, eGFR	8
Littbarski 2018 (long-term) (22)	Germany	Retrospective cohort	Single	67/56	7 years	KDIGO stage, eGFR	8
Oruc 2021 (24)	Turkey	Retrospective cohort	Single	12/15	~5 years	Graft loss, DGF, eGFR	6
van der Plas 2019 (25)	Netherlands	Retrospective cohort	Multicenter	102/83	5 years	eGFR, complications	8
Okada 2019 (26)	Japan	Retrospective cohort	Single	55/53	~5 years	eGFR, hypocalcemia	8
Wang 2023 (27)	USA	Retrospective cohort	Single	23/75	5 years	Graft loss, DGF, Cr	8
Foote 2025 (28)	USA	Retrospective cohort	Multicenter	1,972/541	30 days	30-day morbidity, MACE, readmission	9

### 3.3 Patient characteristics and risk of bias

A total of 4355 patients were enrolled. Among them, 3295 patients underwent PTx were stratified into two core groups: pre-KTx PTx (PTx performed prior to KTx) and post-transplant PTx (PTx performed after KTx). Two studies (Callender et al. (5); Oruc et al. (25)) additionally set a no-PTx control group. Detailed demographic and biochemical parameters at the time of PTx and KTx were documented in **Tables 2**, **3**, respectively.

**Table 2:** Age, PTH and serum calcium at PTx and KTx in the pre-transplant PTx group.

Study	PTH(pg/ml)	Ca(mg/dl)	Pre-PTx(age)	PTH(pg/ml)	Ca(mg/dl)	KTx(age)
Jeon 2012 (23)	1676.5***	10.67 ± 1.22	37.1 ± 11.4	437.2***	9.4 ± 1.2	39.8 ± 12.0
Littbarski 2018 (short-term) (22)	707.6(66.4,2690)*	9.6 ± 0.8	47.0 ± 11.1	–	–	49.0 ± 10.8
Littbarski 2018 (long-term) (22)	760(66,2690)*	9.9 ± 0.9	45.6 ± 12.3	–	–	47.8 ± 12.1
Oruc 2021 (24)	–	–	–	187.5 (11.0-2370.0)*	9.2 (6.3-10.2)*	–
van der Plas 2019 (25)	–	–	–	134.1 (39.6–306.0)*	9.6 (9.0–10.1)*	49 (37.0–60.0)*
Okada 2019 (26)	820.0 (560.0, 1075.0)**	10.4 (10.0, 10.9)**	52.0 (43.0, 58.0)**	53.0 (27.5, 164.0)**	–	56.0 (46.5, 61.0)**
Wang 2023 (27)	1387.8 (881.3–1582.7)**	9.4 ± 1.3	44.8 ± 12.1	89.7 (19.8–196.0)**	9.7 ± 2.3	41.3 ± 12.1

*Median (min – max).

**Median (IQR).

***Detailed data not mentioned in the text.

**Table 3:** Age, PTH and serum calcium at PTx and KTx in the post-transplant PTx group.

Study	PTH(pg/ml)	Ca(mg/dl)	KTx(age)	PTH(pg/ml)	Ca(mg/dl)	Post-PTx(age)
Jeon 2012 (23)	–	–	40.1 ± 10.7	327.3***	11.2 ± 1.0	42.6 ± 9.5
Littbarski 2018 (short-term) (22)	–	–	46.3 ± 12.1	526(111-1513)*	10.8 ± 0.8	46.8 ± 12.0
Littbarski 2018 (long-term) (22)	–	–	47.8 ± 11.1	279(67-1699)*	10.8 ± 0.8	49.5 ± 11.2
Oruc 2021 (24)	1374.0 (293.0-3000.0)*	10 (8.6-12.3)*	–	–	–	–
van der Plas 2019 (25)	591.3 (306.0–1143.0)*	10.4(9.8–10.9)*	38.0 (28.0–54.0)*	–	–	–
Okada 2019 (26)	265.0 (200.0, 428.0)**		51.0 (39.0, 59.0)**	195.0 (147.0, 277.0)**	11.3 (11.1, 11.7)**	52.0 (41.0, 60.0)**
Wang 2023 (27)	642.8 (338.1–1078.4)**	10.1 ± 0.9	51.1 ± 10.8	258.5 (178.0–409.9)**	10.6 ± 0.8	49.5 ± 10.8

*Median (min – max).

**Median (IQR).

***Detailed data not mentioned in the text.

Among 680 patients with available ESKD etiology data, the predominant causes were glomerulonephritis (25.6%), congenital/hereditary nephropathy (14.0%), hypertensive nephropathy (13.5%), and diabetic nephropathy (6.2%). Among the 1842 patients from the seven studies that reported donor type, 38.7% (713/1,842) of patients received living donor kidneys, while 61.3% (1,129/1,842) received deceased donor kidneys.

Data on PTx surgical approaches were documented in 6 cohorts (n=680). Complete subtype classification of PTx procedures was available in 580 patients, whereas surgical details were missing in the remaining 100 individuals.

Among the 580 patients with definitive surgical data, 59.8% (347/580) underwent total PTx with AT, 30.9% (179/580) received subtotal PTx, and 3.1% (18/580) underwent total PTx without AT. The remaining proportion of patients had unreported or other surgical modalities, consistent with the original data from included studies. Detailed surgical information is listed in **Table 4**.

**Table 4:** Surgical modalities of PTx.

Study	Group	Subtotal PTx	Total PTx with AT	Total PTx without AT	Unknown/others
Jeon 2012 (23)	Pre	23	11	–	3
	Post	45	8	–	10
Littbarski 2018 (short-term) (22)	Pre	18	28	6	–
	Post	3	9	2	–
Littbarski 2018 (long-term) (22)	Pre	23	36	8	–
	Post	16	38	2	–
van der Plas 2019 (25)	Pre	25	57	–	20
	Post	38	36	–	9
Okada 2019 (26)	Pre	0	55	–	–
	Post	0	53	–	–
Wang 2023 (27)	Pre	1	15	–	7
	Post	55	20	–	–
Total (580 classifiable)		179 (30.9%)	347 (59.8%)	18 (3.1%)	36 (6.2%)

### 3.4 Risk of bias assessment

The Newcastle-Ottawa Scale (NOS) was applied to evaluate the methodological quality of the included studies. Total NOS scores ranged from 6 to 8 (out of a maximum of 9), suggesting moderate to high methodological quality. All studies achieved satisfactory scores in cohort selection and outcome ascertainment domains, while quality limitations were mainly derived from insufficient adjustment for potential confounding factors in the comparability domain. The detailed NOS scoring results are presented in **Supplementary Table 1**.

### 3.5 Primary outcome graft loss

Three studies reported extractable event data for graft loss (Oruc et al. (25), Okada et al. (27), Wang et al. (28)). The event rates were generally low in both groups: pre-transplant PTx group had 1/12 (8.3%), 3/55 (5.5%), and 2/23 (8.7%), respectively, while the post-transplant PTx group had 3/15 (20.0%), 3/53 (5.7%), and 8/75 (10.7%). Callender et al. (5) reported an adjusted odds ratio of 0.547 (95% CI: 0.327–0.913) favoring pre-transplant PTx. Due to the small number of events and heterogeneity in outcome definitions, no meta-analysis was performed. Overall, available data suggest a potential trend toward lower graft loss with pre-transplant PTx, but evidence remains limited due to low event rates and heterogeneity.

### 3.6 Secondary outcomes

#### 3.6.1 THPT/persistent hyperparathyroidism

Definitions of THPT varied across studies; a qualitative summary is provided in **Table 5**. In Wang et al. (28), persistent hyperparathyroidism (>6 months after PTx) occurred in 1/23 (4.3%) pre-transplant PTx patients vs. 4/75 (5.3%) post-transplant PTx patients. Jeon et al. (24) reported that 36% of pre-transplant PTx patients still had iPTH >65 pg/mL at 1 year post-KTx. Okada et al. (27) did not directly report THPT but found significantly higher serum calcium levels and lower hypocalcemia rates in the pre-transplant PTx group (see below). Due to heterogeneity in definitions and missing data, no pooling was attempted.

**Table 5:** THPT/persistent hyperparathyroidism.

Study	Definition	Pre-transplant PTx	Post-transplant PTx	Notes
Wang 2023 (27)	Persistent HPT (>6 months after PTx)	1/23 (4.3%)	4/75 (5.3%)	No statistical difference
Jeon 2012 (23)	iPTH >65 pg/mL at 1 year post-KTx	12/37 (36%)	Not reported	Still elevated in pre-PTx group
Okada 2019 (26)	Not directly reported	–	–	Only shows higher Ca and less hypocalcemia in pre-PTx group
van der Plas 2019 (25)	Post-transplant HPT recurrence	3.7%	2.3%	No significant difference (P = 0.36)
Callender 2017 (5)	Persistent HPT (non-operated)	–	–	Emphasizes PTH ≥6×ULN associated with graft failure

#### 3.6.2 Estimated glomerular filtration rate

Data on eGFR at different time points were available from Okada et al. (27) and van der Plas et al. (26). Because the two studies used different summary statistics (median with IQR vs. mean ± SEM) and reported different time points, results are presented narratively and in **Table 6**. In Okada et al. (27), pre-transplant PTx was associated with a lower eGFR at 1 month (37.7 vs. 48.6 mL/min/1.73 m², P = 0.002), but this difference disappeared by 36 months (42.5 vs. 42.2 mL/min/1.73 m², P = 0.756). van der Plas et al. found no significant difference in eGFR at 5 years (44.5 ± 4.0 vs. 40.0 ± 6.4 mL/min/1.73 m², P = 0.43).

**Table 6:** Estimated glomerular filtration rate (eGFR) over time.

Study	Time point	Pre-transplant PTx eGFR (mL/min/1.73 m²)	Post-transplant PTx eGFR	P value
Okada 2019 (26)	1 month	37.7 (28.3–47.0)	48.6 (38.1–58.7)	0.002
	6 months	42.2 (36.3–49.2)	46.6 (40.7–51.0)	0.062
	12 months	43.2 (38.7–48.8)	47.6 (39.7–55.0)	0.118
	24 months	41.9 (35.5–47.5)	44.2 (34.6–51.0)	0.502
	36 months	42.5 (34.6–48.0)	42.2 (32.0–47.7)	0.756
van der Plas 2019 (25)	5 years	44.5 ± 4.0	40.0 ± 6.4	0.430

#### 3.6.3 Serum creatinine

Several studies reported serum creatinine (Cr) instead of eGFR; these data are summarized in **Table 7**. In Wang et al. (28), no significant differences in Cr were observed between groups at any time point. Jeon et al. (24) provided limited statistical details, but graphical data suggested similar findings. Littbarski et al. (23) reported Cr in µmol/L; after conversion to mg/dL, no substantial difference was detected. Overall, the serum creatinine results are consistent with the eGFR findings, showing no long-term difference in graft function between the two PTx timing strategies.

**Table 7:** Serum creatinine (Cr) over time.

Study	Time point	Pre-transplant PTx Cr (mg/dL)	Post-transplant PTx Cr (mg/dL)	P value
Wang 2023 (27)	6 months	1.6 ± 0.5	1.5 ± 0.4	0.888
	1 year	1.6 ± 0.5	1.5 ± 0.4	0.704
	2 years	2.1 ± 1.7	1.6 ± 0.6	0.225
	3 years	2.0 ± 1.5	1.6 ± 1.0	0.313
	4 years	1.6 ± 0.7	1.5 ± 0.5	0.608
	5 years	1.5 ± 0.5	1.6 ± 0.6	0.556
Jeon 2012 (23)	1 year	1.38 ± 0.92	1.40 ± 0.62	Not provided

#### 3.6.4 Delayed graft function

Two studies reported DGF. In Wang et al. (28), DGF occurred in 2/23 (8.7%) pre-transplant PTx vs. 15/75 (20.0%) post-transplant PTx; in Oruc et al. (25), DGF was 6/12 (50%) vs. 2/15 (13.3%). No pooling was performed due to small sample sizes and opposite directions of effect.

#### 3.6.5 Thirty-day morbidity and cardiovascular events

One large database study using NSQIP data (Foote et al.) reported 30-day outcomes in 1,972 pre-transplant PTx and 541 post-transplant PTx patients. After propensity score weighting, pre-transplant PTx was associated with higher composite morbidity (13.8% vs. 7.8%; aOR 1.72, 95%CI: 1.13–2.61), higher major adverse cardiovascular events (MACE: 2.2% vs. 0.4%; aOR 8.39, 95%CI: 1.13–62.18), and higher readmission (16.1% vs. 8.1%; aOR 2.07, 95%CI: 1.38–3.10). Mortality and infectious complications did not differ significantly.

### 3.7 Qualitative synthesis of other clinical outcomes

Postoperative hypocalcemia was reported in two studies (27, 28) with inconsistent results: Wang et al. (28) observed a higher hypocalcemia rate in the pre-KTx PTx group (47.8% vs. 17.3%), while Okada et al. (27) found the opposite trend (14.5% vs. 34.0%). Other perioperative complications, including hematoma and transient hoarseness, occurred in less than 5% of patients, with no consistent intergroup discrepancies. Okada et al. (27) reported a markedly higher rate of renal allograft calcium deposition in the post-transplant PTx group (51.1% vs. 7.4%). Similarly, Wang et al. (28) found that the incidence of extraskeletal calcifications was significantly higher in the post-transplant PTx group (21.3% vs. 8.7%).There were no significant intergroup differences in acute rejection rates across all relevant studies (25, 27, 28). Most included studies showed numerically lower rates of graft failure and all-cause mortality in the pre-KTx PTx group, but these differences did not reach consistent statistical significance.

## 4 Discussion

This systematic review of eight studies involving 4355 patients compared pre-transplant versus post-transplant PTx in kidney transplant candidates with severe SHPT. Our main findings are: (i) pre-transplant PTx was associated with a numerically lower risk of graft loss in three studies that reported event data, and one large study reported an adjusted odds ratio favoring pre-transplant PTx (OR 0.547); however, the evidence is limited by small event numbers; (ii) data on tertiary hyperparathyroidism (THPT) were inconsistent due to varying definitions, with no clear advantage for either timing; (iii) although one-month eGFR was lower in the pre-transplant PTx group in one study, this difference disappeared by 36 months, and other studies using serum creatinine showed no long-term difference in graft function; (iv) complication rates (hypocalcemia, hoarseness) varied but were generally manageable; and (v) international guidelines recommend PTx before or early after transplantation, with total PTx with AT or subtotal PTx as preferred techniques. Notably, pre-transplant PTx carries higher short-term risks, including 30-day composite morbidity, MACE, and readmission. This discrepancy may be partially explained by potential selection bias: all kidney transplant recipients are screened to be physically fit for major surgery, while patients undergoing PTx during dialysis often have a heavier comorbidity burden. Differences in baseline health status contribute to the higher complication rates observed in the pre-transplant PTx group. Therefore, decisions should balance long-term graft protection against immediate perioperative safety, rather than favoring pre-transplant PTx unconditionally.

### 4.1 Impact of SHPT on KTx

SHPT directly affects graft outcomes. Callender et al. (5) found that pre-transplant PTH ≥6 times the upper limit of normal was associated with graft failure, and pre-transplant PTx reduced this risk (OR 0.547). Roodnat et al. (6) reported that elevated pre-transplant PTH doubled the risk of graft failure, partly through vascular calcification. Pre-transplant PTH is also an independent predictor of THPT (30, 31), which threatens graft survival (32). Indirectly, SHPT exacerbates pre-existing diabetes via intracellular calcium effects on insulin release (33), and is associated with new-onset diabetes after transplantation (34, 35).

### 4.2 Drugs versus surgery

Pharmacological treatment (vitamin D analogs, calcimimetics) avoids surgical risks but has higher long-term costs (36) and may mask nodular hyperplasia, leading to medically uncontrolled THPT (14, 15). Of note, significant prescription bias exists in the clinical application of cinacalcet among hemodialysis patients: this agent is preferentially prescribed to patients with more severe or refractory hyperparathyroidism. Thus, the adverse outcomes observed in cinacalcet users are largely attributed to baseline disease severity rather than the intrinsic toxicity of the drug itself (37). In addition, calcimimetic use after kidney transplantation may bring a potential risk of hypercalciuria. Although relevant clinical evidence is still limited, this adverse effect is physiologically plausible and has been validated in preclinical experimental studies (38). Surgery provides rapid electrolyte correction and reduces complications including stroke, cardiovascular disease, and fractures (16, 39). PTx reduces cardiovascular events and mortality in ESKD patients (39, 40), restores normocalcemia and PTH, increases femoral neck BMD, and is more cost-effective long-term (16). PTx also improves hemoglobin (41) and cognitive function (42) in long-term dialysis patients. However, the EVOLVE trial showed that cinacalcet’s mortality benefit was confined to patients aged ≥65 years (43). A daily cinacalcet dose >30 mg before KTx was associated with post-KTx re-application, PTx, and bone disease (44). Cinacalcet may mask severe SHPT leading to THPT (45) and its pre-KTx use is linked to nodular hyperplasia (15). Patients on dialysis >3 years who receive cinacalcet are more likely to develop THPT (14). Given a median waiting time of about 4.05 years for a deceased donor kidney in the US (2021-2022) (46), more aggressive SHPT treatment may be needed to optimize use of limited renal grafts. A large cohort study (47) found PTx more effective than cinacalcet for THPT, with PTx within 6 months of KTx improving long-term outcomes. A meta-analysis (48) confirmed that neither PTx nor cinacalcet negatively impacts graft function, but heterogeneity and lack of guidelines limit conclusions to qualitative synthesis.

While pre-transplant PTx appears beneficial for long-term graft survival, the short-term safety profile requires attention. The large NSQIP analysis by Foote et al (29) demonstrated significantly higher 30-day morbidity and MACE after pre-transplant PTx. These findings underscore the need for individualized decision-making, weighing the immediate perioperative risks against the potential long-term graft protection.

### 4.3 International discrepancies in guidelines and clinical practice

PTH control targets and surgical indications vary across countries. Japan has adopted a more aggressive approach, with lower PTH targets (60–240 pg/mL) associated with higher survival rates in hemodialysis patients despite a low KTx rate (40). Nodular hyperplasia is suspected when PTH >800 pg/mL or parathyroid glands >1 cm or >500 mm³ on ultrasound, often requiring surgery (40). The Japanese Society for Dialysis Therapy recommends pre-KTx PTx for advanced SHPT (49).

Despite international variations in PTH thresholds, guidelines from the regions represented in included studies (American, European, German, and Japanese) consistently recommend that PTx be performed either before or early after KTx for patients with severe SHPT, with total PTx with AT or subtotal PTx as the preferred surgical approaches (39, 50–52). The details are presented in **Supplementary Table 3**. It is well documented that serum PTH levels tend to decline spontaneously within the first year following kidney transplantation (7, 53). This phenomenon is a key reason why many clinicians and guidelines recommend delaying parathyroidectomy for at least one year after transplantation. However, these recommendations are largely based on observational evidence and expert consensus, lacking high-quality quantitative synthesis that directly compares pre-transplant with post-transplant PTx.

Our systematic review addresses this evidence gap by providing a comprehensive narrative synthesis suggesting that pre-transplant PTx may be associated with lower graft loss and comparable long-term renal function. Most included studies support pre-KTx PTx for better graft outcomes. For example, Callender et al. (5) reported an adjusted odds ratio of 0.547 (95% CI: 0.327–0.913) for graft failure favoring pre-transplant PTx, and the three studies with extractable event data all showed numerically lower graft loss rates in the pre-transplant PTx group. Although data on THPT were inconsistent due to varying definitions, five of the eight included studies (Jeon et al. (24), Okada et al. (27), Wang et al. (28), Callender et al. (5), Oruc et al. (25)) concluded that PTx should ideally be performed before KTx. Thus, our findings support current guideline recommendations and strengthen the evidence base for favoring pre-transplant PTx in suitable kidney transplant candidates, particularly with respect to graft survival and long-term renal function.

### 4.4 A proposed risk-stratified clinical algorithm

Based on the synthesized evidence and accounting for inter-country differences in kidney waitlist times, we propose a risk-stratified algorithm to guide PTx timing in kidney transplant candidates. Our framework explicitly incorporates waitlist time as a novel and critical decision node, recognizing that waiting periods vary dramatically across regions (e.g., >4 years in the US vs. short intervals in living-donor-dominant Japan (46)).

The algorithm integrates four evidence-based dimensions:

Estimated waitlist time – Based on published data (46) and clinical practicality, we propose three illustrative categories: >2 years (prolonged, favoring pre-transplant PTx), 1–2 years (intermediate, requiring individualized assessment), and <1 year (short, allowing post-transplant observation). These thresholds are not fixed but should be adapted to local waitlist dynamics.

PTH level and parathyroid morphology – Nodular hyperplasia, which is often refractory to medical therapy, is suspected when PTH >800 pg/mL for >6 months or when parathyroid glands exceed 1 cm in diameter (or volume ≥500 mm³) on ultrasound (18, 40). These thresholds are supported by multiple guidelines and the roadmap proposed by Cianciolo et al. (18).

Dialysis vintage – In the included studies, patients who underwent pre-transplant PTx had longer dialysis duration, indicating that prolonged dialysis exposure is common in this population. Based on these data and the established relationship between dialysis vintage and parathyroid hyperplasia (18, 40), a dialysis vintage >5 years is considered suggestive of established nodular hyperplasia and reduced responsiveness to calcimimetics.

Predicted risk of persistent hyperparathyroidism after KTx – A validated nomogram (54) (Ma et al., AUC 0.926) can quantify individual risk using dialysis duration, postoperative 3-month iPTH, corrected calcium, and phosphorus. This tool further refines decision-making, particularly in patients with intermediate-risk features.

Based on the combination of these factors, patients are directed toward one of three management pathways: pre-transplant PTx (for those with long waitlist time, high PTH/nodular hyperplasia, or high nomogram risk), early post-transplant PTx (≤12–18 months) for those with moderate risk but short waitlist time, or medical therapy (for low-risk patients with anticipated spontaneous resolution or those unfit for surgery).

This framework moves beyond “one-size-fits-all” recommendations and provides a clinically actionable, resource-aware decision tool. It should be validated prospectively and adapted to local transplant logistics.

### 4.5 Surgical modalities and complications

Multiple surgical approaches were applied in the eligible studies, among which total PTx with AT and subtotal PTx were predominant. The included studies did not share a consistent definition of subtotal parathyroidectomy, and details on gland resection and residual tissue volume were rarely documented. According to clinical published single-center data (5), this procedure refers to resecting three entire parathyroid glands while leaving 20–30 mg of vascularized parathyroid tissue *in situ* to prevent permanent hypoparathyroidism. Compared with subtotal PTx or total PTx with AT, total PTx without AT carries a higher risk of reoperation for recurrent SHPT and more severe hypoparathyroidism (55–58).

Cervical ultrasound and 99mTc-MIBI scintigraphy were the common preoperative localization tools mentioned in several studies. Nevertheless, no data regarding ectopic parathyroid glands were available from the included articles. Accurate preoperative localization (ultrasound, 99mTc-MIBI, SPECT/CT) (59) and intraoperative PTH measurement (60) are essential to reduce recurrence. Ablation therapy (thermal, chemical) is emerging as a minimally invasive alternative for patients with poor cardiorespiratory fitness (61–64), though its role in KTx candidates requires further study.

### 4.6 Limitations and future directions

Several limitations should be acknowledged. First, all included studies were retrospective, with inherent selection bias and confounding by indication. Second, outcome definitions (e.g., THPT, hypocalcemia) varied across studies, precluding meta-analysis. Third, PTH assays and surgical techniques were not uniform. Fourth, sample sizes were relatively small, especially in the pre-transplant PTx groups. Fifth, publication bias cannot be excluded, though we searched multiple databases and grey literature. Prospective randomized trials are needed to validate optimal PTx timing and to test the proposed risk-stratified algorithm that incorporates waitlist time, PTH level, dialysis vintage, and predictive nomograms. Sixth, the large database study (Foote et al. (29)) had only 30-day follow-up and did not provide long-term graft outcomes, and its population may partially overlap with other included studies, though this could not be quantified.

## 5 Conclusion

KTx does not resolve all SHPT. Our systematic review suggests that pre-transplant PTx may be associated with reduced graft loss and comparable long-term renal function compared with post-transplant PTx, although the evidence is limited by small event numbers and heterogeneous outcome definitions. However, pre-transplant PTx carries higher short-term risks, including 30-day morbidity and major adverse cardiovascular events. Therefore, the decision should individualize the trade-off between long-term graft protection and immediate perioperative safety. The proposed risk-stratified algorithm, incorporating waitlist time, PTH level, dialysis vintage, and a validated nomogram, may help guide this decision. Prospective studies are needed to validate this framework.

## Data Availability

The original contributions presented in the study are included in the article/**Supplementary Material**. Further inquiries can be directed to the corresponding author.
