# Supplementary material for: Timing of parathyroidectomy in kidney transplant candidates: a systematic review and a proposed clinical algorithm
Source: Front Endocrinol (Lausanne). 2026 Jul 3;17:1871106. doi: 10.3389/fendo.2026.1871106 (PMC13375521; doi:10.3389/fendo.2026.1871106)
Supplement: Supplementary file 1 [file Table1.doc]

Supplementary Table S1. Newcastle‑Ottawa Scale (NOS) scores

| Study (year) | Selection (max 4★) | Comparability (max 2★) | Outcome (max 3★) | Total (max 9★) |
| --- | --- | --- | --- | --- |
| Callender 2017 | ★★★★ | ★★ | ★★ | 8 |
| Jeon 2012 | ★★★ | ★ | ★★ | 6 |
| Littbarski 2018 (short‑term) | ★★★★ | ★★ | ★★ | 8 |
| Littbarski 2018 (long‑term) | ★★★★ | ★★ | ★★ | 8 |
| Oruc 2021 | ★★★ | ★ | ★★ | 6 |
| van der Plas 2019 | ★★★★ | ★★ | ★★ | 8 |
| Okada 2019 | ★★★★ | ★★ | ★★ | 8 |
| Wang 2023 | ★★★★ | ★★ | ★★ | 8 |
| Foote 2026 | ★★★★ | ★★ | ★★★ | 9 |

Supplementary Table S2. Raw graft loss events

| Study | Pre‑transplant PTx (events/total) | Post‑transplant PTx (events/total) |
| --- | --- | --- |
| Oruc 2021[24] | 1/12 (8.3%) | 3/15 (20.0%) |
| Okada 2019[26] | 3/55 (5.5%) | 3/53 (5.7%) |
| Wang 2023[27] | 2/23 (8.7%) | 8/75 (10.7%) |
| Callender 2017[5] | aOR 0.547 (0.327–0.913) (Reference) | |

Supplementary Table S3. Recommendations for parathyroidectomy for kidney transplantation in different countries or regions

| **Professional Organization** | **Year Published** | **PTx-Related Recommendations for Kidney Transplantation** |
| --- | --- | --- |
| American Association of Endocrine Surgeons | 2022 | Referral for PTX for THPT should be placed within 12 months postkidney transplant.  Total PTX without autotransplantation should not be performed on patients with CKD who may undergo kidney transplantation. |
| European Society of Endocrine Surgeons | 2015 | Level 4 evidence favours PTX prior to renal transplantation in with persistent or high probability of developing renal HPT. |
| German Association of Endocrine Surgeons | 2021 | For symptomatic patients with rHPT listed for renal transplantation, subtotal parathyroidectomy should be considered.  Severe symptomatic hypercalcemia in the first months after renal transplantation should be managed by a multidisciplinary team, indicating early subtotal parathyroidectomy. |
| Japanese Society for Dialysis Therapy | 2013 | We recommend that bone and mineral metabolism should be adequately managed before kidney transplantation in order to achieve good control of mineral metabolism after transplantation.  For patients with hypercalcemia (corrected calcium ≥10.5 mg/dL) and/or those with elevated PTH (greater than the upper limit of the reference range at the facility) ≥1 year after transplantation, we suggest considering parathyroid intervention. |
